# Prevalence of frailty in Japan: A systematic review and meta-analysis

**DOI:** 10.1016/j.je.2016.09.008

**Published:** 2016-11-15

**Authors:** Gotaro Kojima, Steve Iliffe, Yu Taniguchi, Hiroyuki Shimada, Hiromi Rakugi, Kate Walters

**Affiliations:** a Department of Primary Care and Population Health, University College London, London, United Kingdom; b Research Team for Social Participation and Community Health, Tokyo Metropolitan Institute of Gerontology, Tokyo, Japan; c Section for Health Promotion, Center for Gerontology and Social Science, National Center for Geriatrics and Gerontology, Aichi, Japan; d Department of Geriatric and General Medicine, Osaka University Graduate School of Medicine, Osaka, Japan

**Keywords:** Frailty, Prevalence, Japan, Community-dwelling older people

## Abstract

Japan's population is aging more rapidly than that of any other country. Frailty has recently been recognized as an important priority. Understanding the basic epidemiology of frailty in Japan, which is an example of a rapidly aging society, will be beneficial for Japan as well as other countries expecting an aging population. A systematic literature search of 11 electronic databases was conducted in March 2016 using a comprehensive set of Medical Subject Heading and text terms for any studies published in 2000 or later that report the prevalence of frailty among Japanese community-dwelling older people aged 65 years or older. A total of 1529 studies were identified in the systematic search, of which five studies were included in this review. The pooled prevalence of frailty, prefrailty, and robustness was 7.4% (95% confidence interval [CI], 6.1%–9.0%), 48.1% (95% CI, 41.6%–54.8%), and 44.4% (95% CI, 37.2%–51.7%), respectively. A significant degree of heterogeneity was observed. There was no evidence of publication bias. Age-stratified meta-analyses of four studies showed the pooled prevalence of frailty was 1.9%, 3.8%, 10.0%, 20.4%, and 35.1% for those aged 65–69, 70–74, 75–79, 80–84, and ≥85 years, respectively. Pooled prevalence of frailty was 8.1% for women and 7.6% for men. This review showed an overall pooled prevalence of frailty among Japanese community-dwelling older people of 7.4%. The age-stratified analysis suggested that Japanese older people are less frail before their late 70's but frailer in later life than older people in other countries. These findings provide important basic information for all parties involved in Japanese frailty research.

## Highlights

• Frailty is an important priority in Japan, where the population is aging rapidly.
• Japan is a good example of a rapidly aging society for frailty research.
• Five studies were included in this systematic review and meta-analysis study.
• Pooled prevalence of frailty in Japanese in the community aged >65 years is 7.4%.

## Introduction

Because Japan has the world's highest life expectancy and a persistently low birth rate, Japan's population is aging more rapidly than that of any other country.^1^ The proportion of people aged 65 years and older was approximately 10% in 1985. This proportion doubled to 21% in 2007, making Japan a hyper-aged society.^2^, ^3^ The latest governmental provisional estimates report that as much as 26.7% of the Japanese population were over 65 years old in 2015.^4^ This rate is much higher than in other developed countries: 17% in the United Kingdom, 14% in the United States, 9% in China, 21% in Germany, 19% in France, 22% in Italy, and 16% in Canada.^3^ By 2060, Japan is expected to far exceed the current understanding of a hyper-aged society, when 40% of the entire population will be aged 65 years or over.^5^ With the increasing number of older people in Japan, there is expected to be a substantial increase in health care and social security costs.

The Japanese government has already begun to prepare for this challenging issue.^6^ It has started to attempt to adapt society in order to maximize older people's health and to facilitate healthy aging via maintaining their functional capacity and preventing disability and dependence.^7^ One of the campaign targets is frailty.^8^ Frailty is a state of vulnerability to poor resolution of homeostasis when exposed to a stressor event as a consequence of age-related cumulative deficits across multiple physiological systems.^9^ It is considered a pre-disability state and is associated with various negative health outcomes, including falls, hospitalization, institutionalization, fracture, disability, dementia, lower quality of life, and mortality.^9^, ^10^, ^11^, ^12^, ^13^, ^14^, ^15^, ^16^ The most widely used definition of frailty is the frailty phenotype proposed by Fried et al. using data from the Cardiovascular Health Study.^17^ They recognized frailty as a distinct clinical syndrome using a set of five physical phenotypic components: unintentional weight loss, exhaustion, weakness, slow walking speed, and low physical activity with an underlying biological basis.^17^ In the Fried criteria, an individual is classified as frail, prefrail, and robust when they meet ≥3, 1–2, and 0 of the components, respectively.^17^

Given the detrimental physical and psychological impact of frailty on older people, as well as its potential reversibility,^18^, ^19^ frailty may be a promising target of interventions.^9^ Frail older people, who are not highly fit but not completely disabled, are the population likely to benefit most from such interventions.^20^ In this regard, frailty can be an important outcome, for which it is worth exploring the modifiable risk factors or predictors to be addressed for prevention.^21^ Therefore, understanding the basic epidemiology of frailty in older people is essential for clinicians, researchers, and policymakers to further pursue frailty research and support.^22^, ^23^

According to previous systematic reviews,^24^, ^25^ the prevalence of frailty based on the Fried criteria among community-dwelling people aged 65 years and older ranged from 4% in a United States study^26^ to 27.3% in a Spanish study.^27^ One of these reviews performed a meta-analysis using data from 15 studies and showed that the weighted prevalence of physical frailty was 9.9%.^24^

In other selected populations, the prevalence of frailty has been reported to be much higher, such as in patients with cancer (range, 6–28%; based on the Fried criteria)^28^ and nursing home patients (pooled prevalence 46.9%; 95% confidence interval [CI], 27.7%–66.6%; based on the Fried criteria).^29^ Japanese studies were not included in the aforementioned reviews, and the evidence regarding the prevalence of frailty among the Japanese population is scarce in the literature. Understanding the current frailty status in Japan as an example of a rapidly aging society will be beneficial for research and health policy for Japan as well as any other countries experiencing rapid population aging.

The purposes of this systematic review and meta-analysis study were two-fold: to systematically search the literature for available evidence on the prevalence of physical frailty among Japanese community-dwelling older people, and to conduct a meta-analysis to synthesize the pooled prevalence of frailty.

In general, people become frailer with age and females are more likely to be frailer than their male counterparts.^24^ However, since Japan is unique in its longevity,^30^ universal health insurance system, healthy Japanese food, enhanced awareness about healthy aging among the general public, and the so-called Japanese smoking paradox (Japanese people smoke more but develop less lung cancer than people in Western countries), Japanese people may have different courses and patterns of frailty status than other populations. In addition, merely pooling the prevalence of frailty without taking into consideration the cohort characteristics, such as mean age or female proportion, may obscure important subgroup differences. Therefore, we also performed meta-analyses stratified according to age and gender.

## Methods

### Protocol

A protocol was developed according to the Preferred Reporting Items for Systematic Review and Meta-Analysis (PRISMA) statement^31^ and has been published elsewhere.^32^

### Data sources and search strategy

A systematic search of the literature was conducted in March 2015 for studies published in 2000 or later by two investigators (GK and YT) using ten electronic databases: Scopus, Web of Science, Embase, MEDLINE, LILACS, CINAHL Plus, PsycINFO, Cochrane Library, AMED, and ICHUSHI Web, with explosion function if available and without language restriction. The search terms used for the databases, except those for ICHUSHI Web, are as follows: [(Frailty) OR (Frailty syndrome (Medical Subject Heading (MeSH)))] AND [(Japan*) OR (Japan (MeSH))]. ICHUSHI Web is a Japanese bibliographic database containing mainly Japanese articles and both English and Japanese terms can be used for searching. The search terms for ICHUSHI Web included (Frailty) and Japanese MeSH and text terms corresponding to frailty, and the studies were limited to original articles involving people aged 65 years or older, excluding case reports or case series. Other data sources included the manual search of bibliographies of the relevant articles, personal inquiries to experts in this field, and a search of another Japanese electronic database, Japan Science and Technology Information Aggregator, Electronic (J-STAGE), using a search strategy similar to the one for ICHUSHI Web. Corresponding and/or last authors were contacted for additional data necessary for a meta-analysis. Ethics approval was not required, as this study did not involve human subjects.

### Study selection and methodological quality assessment

Any studies providing or potentially capable of providing cross-sectional data regarding prevalence of frailty status defined using the Fried criteria or its modified versions among Japanese community-dwelling people aged 65 years and older in Japan were eligible. Studies were excluded if they used selected samples, such as people with certain diseases or conditions, or were a randomized controlled trial, review article, editorial, or comment. Gray literature, such as conference abstracts, was also considered. When multiple studies used the same cohort, the study with the largest sample size was included. A clinician researcher with an internal medicine and geriatric medicine background (GK) assessed the identified studies via screening titles, abstracts, and full texts. The studies considered as eligible were further assessed for methodological quality using six items from a tool for critically appraising studies of prevalence or incidence of a health problem developed by Loney et al.^33^ Studies meeting three or more of the six items were considered to have adequate methodological quality and were included in the meta-analysis.

### Data extraction

The data collected directly from the included studies or provided by the authors upon request were first author's name, cohort name if any, publication year, prefecture where the participants were recruited from, sample size, age (mean and range), proportion of female participants, percentage and the number of participants according to frailty categories (frail, prefrail, and robust), and cohort characteristics.

### Statistical analysis

The numbers of the entire cohort, as well as those classified frail, prefrail, and robust, were used for analysis. Heterogeneity across the studies was assessed using Cochran's Q test, and heterogeneity was considered to be present when p < 0.05. The degree of heterogeneity was assessed using the I^2^ statistic. The I^2^ values of 25%, 50%, and 75% were considered as low, moderate, and high degrees of heterogeneity, respectively.^34^ Pooled prevalence and 95% CIs of frailty, prefrailty, and robustness were calculated using a random-effects model if heterogeneity was present and a fixed-effects model if heterogeneity was absent. Publication bias was assessed using Begg-Mazumdar's^35^ and Egger's^36^ tests and visually inspecting the funnel plots. All statistical analyses were completed using StatsDirect (ver. 2.8, StatsDirect, Cheshire, UK) and p < 0.05 was considered statistically significant.

## Results

### Selection processes

**Fig. 1:**
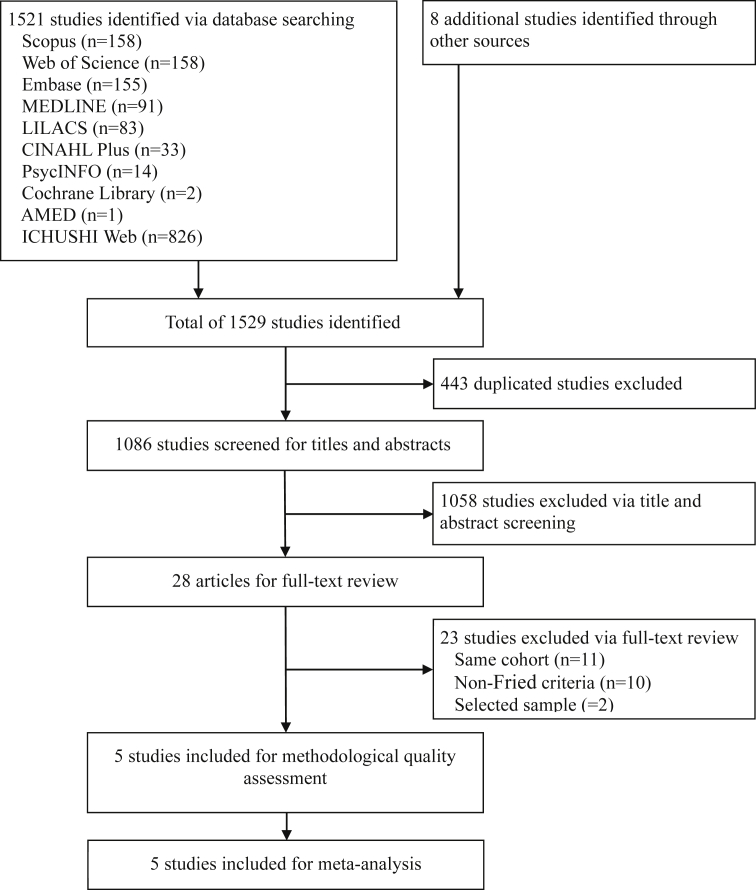
PRISMA flow chart.

The systematic search of the literature using ten electronic databases identified 1521 citations, and eight additional citations were found through other sources. Of these 1529 citations, 443 were duplicates and were removed, and 1058 citations were excluded through title and abstract screening. Twenty eight studies were left for full-text review, of which 23 studies were excluded because they used the same cohort (n = 11), used a method other than the Fried criteria to define frailty (n = 10), or used a selected sample (n = 2), leaving five included studies. The methodological quality of the five studies was assessed and considered to be adequate for meta-analysis (Fig. 1).

### Study characteristics

The characteristics of the five included studies are summarized in Table 1. Four studies^37^, ^38^, ^39^, ^40^ were published as journal articles, and one study^41^ was presented as a poster at a scientific meeting. All studies were published in 2011 or later. Two studies^39^, ^41^ were conducted in prefectures around or near Tokyo (Gunma, Ibaraki, Chiba, and Fukushima Prefectures). The other studies^37^, ^38^, ^40^ were from more western areas (Fukuoka, Aichi, and Kyoto Prefectures). Sample sizes ranged from 483^40^ to 8864.^38^ Mean age was similar among four studies at 73.3–74.3 years,^37^, ^38^, ^40^, ^41^ and one study did not report the mean age or age range.^39^ While one study included only female participants,^41^ the remaining studies were mixed, with approximately 50–70% females.^37^, ^38^, ^39^, ^40^ No studies used cohorts of a nationally representative elderly population. Three studies^37^, ^38^, ^39^ used locally representative cohorts and two used samples originally recruited at health events.^40^, ^41^ Exclusion criteria were provided in three studies,^37^, ^38^, ^40^ which excluded individuals with disability,^38^, ^40^ neurological and cognitive disorders,^37^, ^38^, ^40^ or those using long-term care services.^37^, ^38^, ^40^

**Table 1:** Summary of study characteristics and overall prevalence of frailty status among Japanese community-dwelling older people in Japan^a^.

Author/Study	Year	Prefecture	Sample size	Age (range)	Female (%)	Frail	Prefrail	Robust	Quality score	Cohort characteristics
Shirooka et al.	2016	Kyoto	483	73.3 (65–92)	68.3%	8.3% (40)	65.2% (315)	26.5% (128)	4/6	- Health event - Exclusion criteria: in long-term care service, ADL disability, severe cardiac, pulmonary, neurological, and musculoskeletal disorders, dementia
Chen et al. Sasaguri Genkimon Study	2015	Fukuoka	1565	73.3 (65–93)	60.1%	9.5% (149)	43.9% (687)	46.6% (729)	5/6	- Population-based study - Exclusion criteria: in long-term care service, dementia, Parkinson's disease, depression
Shimada et al. NCGG-SGS	2015	Aichi	8864	73.4 (65–96)	52.0%	8.4% (743)	51.0% (4517)	40.7% (3604)	5/6	- Population-based study - Exclusion criteria: in long-term care service, ADL disability, Parkinson's disease, stroke, depression, dementia, MMSE < 21
Shinkai et al. Kusatsu longitudinal study	2013	Gunma	526	− (≥65)	–	5.7% (30)	38.0% (200)	56.3% (296)	4/6	- Population-based study - Exclusion criteria: not documented
Seino et al.	2011	Ibaraki, Chiba, Fukushima	502	74.3 (65–92)	100%	4.6% (23)	42.7% (214)	52.8% (265)	4/6	- Health event - Exclusion criteria: not documented

NCGG-SGS, National Center for Geriatrics and Gerontology-Study of Geriatric Syndromes.

a All studies used modified Cardiovascular Health Study frailty criteria.

### Prevalence of frailty

**Fig. 2:**
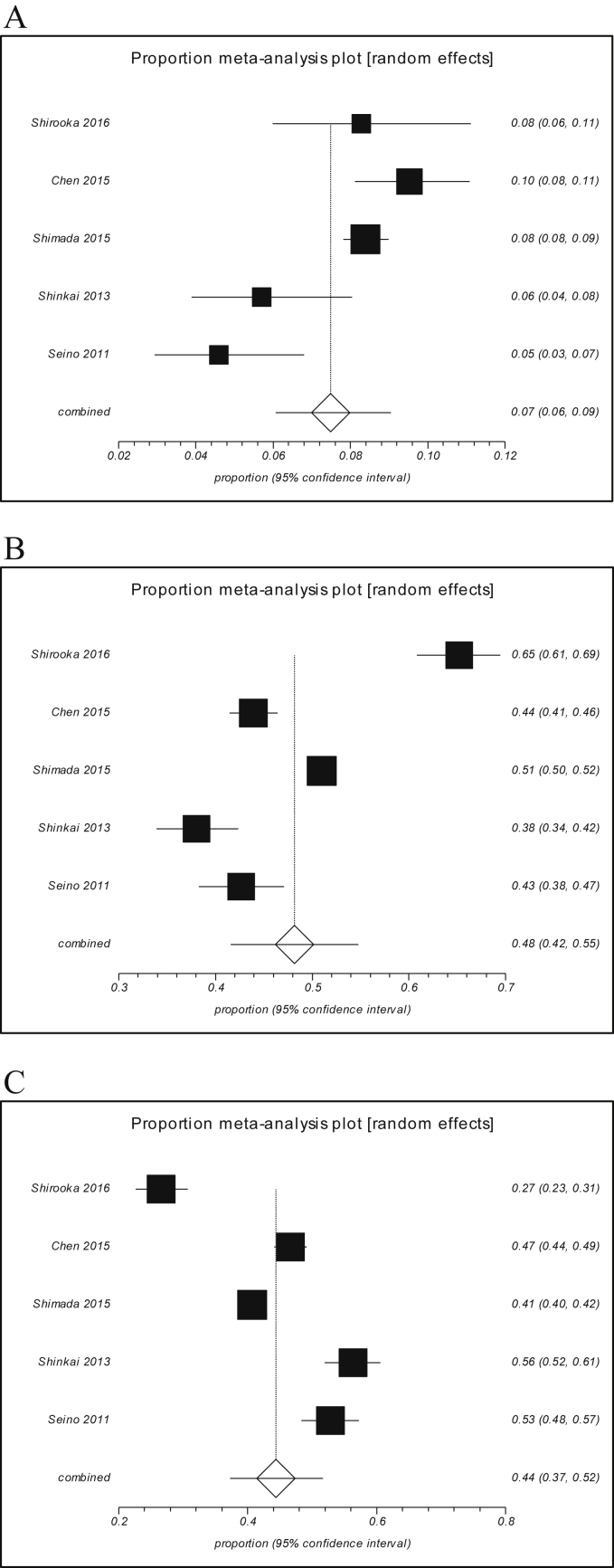
Forest plots of prevalence of (A) frailty, (B) prefrailty, and (C) robustness among Japanese community-dwelling older people in Japan.

Data from five studies were available for meta-analysis of the prevalence of frailty status. The prevalence of frailty, prefrailty, and robustness in individual studies ranged 4.6%–9.5%, 38.0%–65.2%, and 26.5%–56.3%, respectively. Since significant degrees of heterogeneity were observed across the studies for the three frailty categorizations (I^2^ = 79.2%–97.1%, all p < 0.001), random-effects models were used. The pooled prevalences of frailty, prefrailty, and robustness were 7.4% (95% CI, 6.1%–9.0%), 48.1% (95% CI, 41.6%–54.8%), and 44.4% (95% CI, 37.2%–51.7%), respectively (Fig. 2). There was no evidence of publication bias according to Begg-Mazumdar's and Egger's tests (all p > 0.05) and visual inspection of the funnel plots (not shown).

### Stratified analysis

**Fig. 3:**
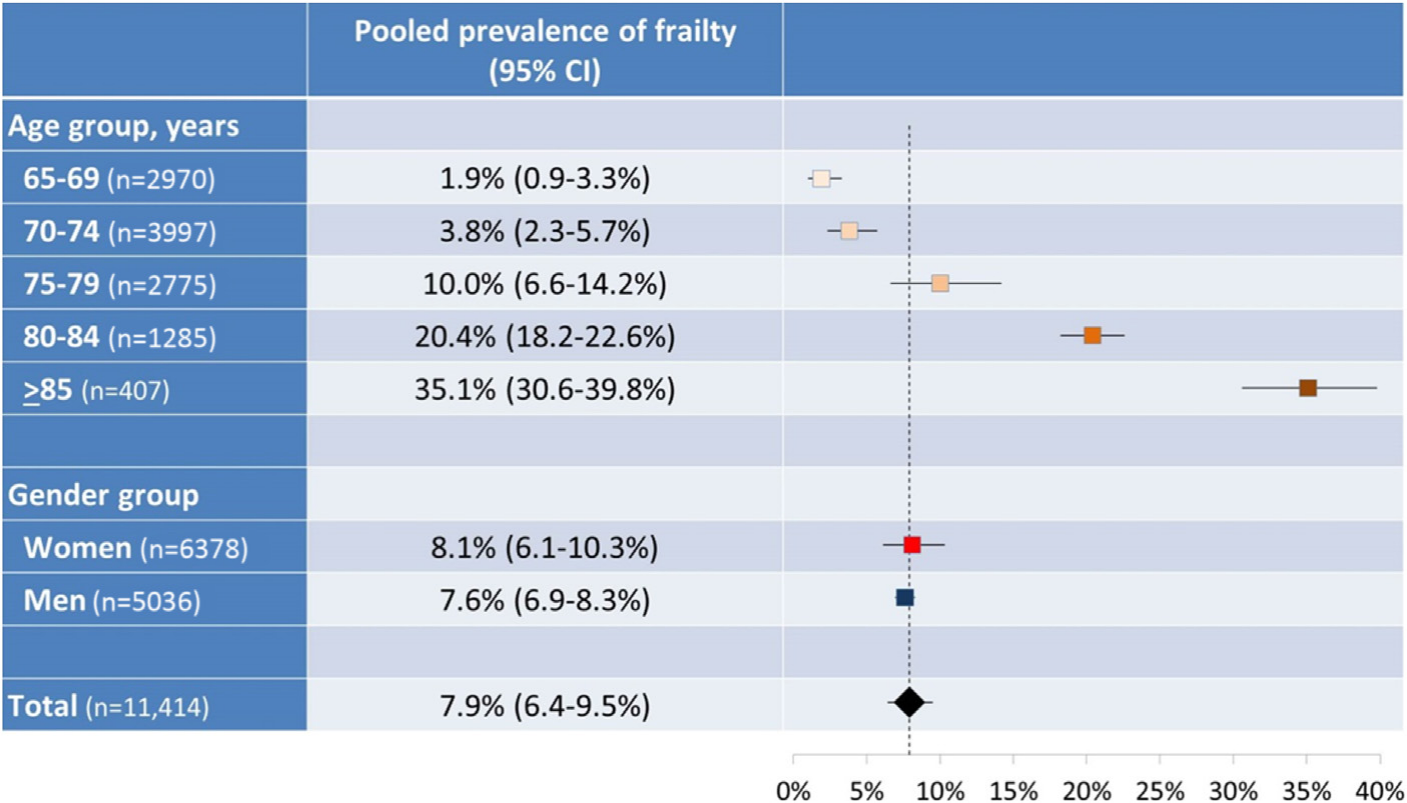
Forest plots of prevalence of frailty stratified by age groups and gender.

Additional data were obtained upon request from the authors of four studies^37^, ^38^, ^40^, ^41^ and were used for meta-analysis stratified by age, gender, and both. The pooled prevalence of frailty in five age groups (65–69, 70–74, 75–79, 80–84, and ≥85 years old) was 1.9%, 3.8%, 10.0%, 20.4%, and 35.1%, respectively (Fig. 3). As one study^41^ included only women, four studies^37^, ^38^, ^40^, ^41^ were used for stratified meta-analysis for women, while three studies^37^, ^38^, ^40^ were used for men. The pooled prevalence of frailty was 8.1% for women and 7.6% for men (Fig. 3). When stratified by both age group and gender, prevalence of frailty increased with age in men and women. More women were frail than men in most age groups, except for 70–74 years old (Table 2).

**Table 2:** Prevalence of frailty stratified by age groups and gender.

Age group, years	Women		Men	
	Prevalence	95% CI	Prevalence	95% CI
65–69	2.1%	0.7–4.3%	1.8%	1.2–2.6%
70–74	3.8%	1.7–6.6%	4.2%	3.3–5.1%
75–79	10.1%	5.9–15.1%	7.7%	1.7–17.6%
80–84	22.3%	19.3–25.4%	18.1%	15.1–21.3%
≥85	37.2%	31.1–43.6%	32.3%	25.8–39.3%

CI, confidence interval.

#### Changes in heterogeneity

A high degree of heterogeneity persisted among women (I^2^ = 81.8%, p < 0.001) and the younger three age groups: 65–69, 70–74, and 75–79 years (I^2^ = 68.6%–81.1%, all p < 0.05), while the heterogeneity decreased to non-significant levels among men (I^2^ = 42.4%, p = 0.18) and in the older two age groups: 80–84 and ≥ 85 years old (I^2^ = 0%–54.2%, all p > 0.05), which suggests that the high heterogeneity may be partially explained via variations in gender and age.

## Discussion

This systematic review and meta-analysis identified five studies incorporating 11,940 Japanese people aged 65 years or older living in the community and demonstrated that the pooled prevalences of frailty, prefrailty, and robustness based on the Fried criteria were 7.4%, 48.1%, and 44.4%, respectively. Stratified analyses showed that women were frailer than men and that prevalence of frailty increased with age.

Two previous systematic reviews^24^, ^25^ reported the prevalence of frailty in multinational community-dwelling elderly populations, and one^24^ of these further performed meta-analysis. Our review included only Japanese older people, and it showed some distinctive and notable findings compared with these previous reviews. First, the range of frailty prevalence reported by the included studies (4.6–9.5%) was much narrower than that of previous reviews (4.0–17.0%^24^ and 4.9–27.3%,^25^ both based on the Fried criteria). This can be explained by the fact that the current review included only Japanese older people in Japan, while the previous reviews included cohorts from multiple different countries; therefore, there may have been less between-individual and between-study variations in the prevalence of frailty in our review than in the previous ones. Another possible explanation is that the included cohorts were similar in terms of age because the mean age and age range of the cohorts were fairly close (mean age ranged from 73.3 to 74.3 years, and included age ranges were nearly identical) (Table 1). Second, our pooled prevalence of frailty based on the Fried criteria (7.5%; 95% CI, 6.1%–9.0%) was lower than weighted prevalence of physically-defined frailty by Collard et al. (9.9%; 95% CI, 9.6%–10.2%).^24^ Among the studies included in our review, some recruited participants from health events^40^, ^41^ or a health check-up^39^ or excluded those with activities of daily living (ADL) disability,^38^, ^40^ which may have contributed to a lower pooled prevalence of frailty than that of the general elderly population. It should be noted that our review and that of Collard et al.^24^ used different statistical methodologies and included studies using different frailty criteria. Third, stratified meta-analyses were performed to further examine the pooled prevalence of frailty according to age and gender. The result of the gender-stratified analysis showing that women were frailer than men was not surprising and was in line with previous reports.^24^ As also expected, the age-stratified analysis showed that the prevalence of frailty increased steadily with age. In a previous review,^24^ age-stratified weighted prevalence of frailty (based on any frailty definition) among community-dwelling older people from multiple countries was approximately 4% in those aged 65–69 years; 7% in those aged 70–74 years; 10% in those aged 75–79 years; 16% in those aged 80–84 years; and 26% in those aged ≥85 years. Compared with these findings, our review showed that the younger two age groups had lower prevalence (1.9% in those aged 65–69 years and 3.8% in those aged 70–74 years), the 75–79-year age group had the same prevalence (10.0%), and older age groups had higher prevalence (20.4% in those aged 80–84 years and 35.1% in those aged ≥85 years). The reasons why the prevalence of frailty was lower in the young elderly and higher in the older elderly in our study are not clear. One possibility, given the high life expectancy among Japanese, is that non-Japanese frail older people die early, which leads to lower prevalence of frailty in later years due to the heathy survivor effect,^42^ while Japanese frail older people survive longer, which leads to higher prevalence of frailty in their 80's and later. Further research regarding the impact of environmental factors and ethnic background on frailty status and progression may be helpful to elucidate this discrepancy.

There are two tools that have been developed and used in Japan for identifying vulnerable older adults with high risks not specifically for frailty but for general adverse health outcomes, such as dependency, disability, or institutionalization.

The Kihon Checklist (KCL) is a self-reported comprehensive questionnaire consisting of 25 simple questions covering multiple domains of instrumental ADL, physical function, oral function, nutrition, cognition, social activity, and depressive mood. The checklist was originally developed by the Japanese Ministry of Health, Labour and Welfare in 2005–2006, when the long-term care insurance system, which had originally been started in 2000, was reformed to focus more on prevention.^43^ This questionnaire has been widely used in Japanese local municipal offices and centers as an initial screening tool to identify at-risk older individuals and, if necessary, initiate interventional programs and facilitate various governmental long-term care and support services according to their conditions. This tool was validated as a screening tool for frailty and shown to have good-to-excellent accuracy: the area under the receiver operating characteristic curve (AUC) to predict frailty (defined by the Fried criteria) was 0.92 in a sample of geriatric outpatients with chronic diseases^44^ and 0.88 in a sample of community-dwelling older people.^45^ One study involving 14,636 Japanese elderly aged 65 years or older identified 38.0% as frail based on the KCL; frail participants were significantly more likely to be newly certified for long-term-care insurance over 1 year than those who were non-frail (OR 3.80; 95% CI, 3.02–4.78).^46^

Another tool is the Frailty Index for Japanese elderly (FI-J), also known as the Kaigo-Yobo Checklist, which is a 15-item questionnaire to identify older adults at high risk of becoming dependent or in need of long-term care.^47^ This index has also been validated for frailty screening in a population-based study, with good accuracy to predict Fried criteria-defined frailty (AUC = 0.89), and frailty defined by this index was a significant predictor of incident difficulties in ADL (OR 3.42; 95% CI, 1.79–6.54 over 4 years), incident long-term-care certification (HR 3.50; 95% CI, 2.41–5.07 over 5 years), and mortality (HR 2.43; 95% CI, 1.70–3.47 over 5 years) independent of age, gender, and presence of comorbidity.^39^

These feasible self-report questionnaires are easy to implement and have potential as frailty screening tools covering multidimensional components. Now that both have been shown to identify, with good-to-excellent accuracy, Fried-defined frail older people, future research can be designed to compare their abilities to predict important outcomes, such as mortality or the long-term-care need, with other widely used frailty criteria, or to use these tools as a continuous index similar to the Rockwood Frailty Index,^48^ instead of the two-group dichotomization (frailty vs. non-frailty), in order to make the most of the nature as a continuous score to evaluate and capture frailty status in a graded manner. These attempts may lead to the discovery of the best frailty measure suitable for Japanese community-dwelling older people.

The findings of the current review need to be interpreted with caution. First, the pooled prevalence of frailty synthesized from the included studies may be smaller than the true prevalence. As mentioned earlier, some of the included studies recruited older people at health events^40^, ^41^ or a municipal annual health check-up,^39^ and some studies excluded those with ADL disability.^38^, ^40^ Therefore, the population included in the meta-analysis may be more health conscious and/or less disabled, possibly resulting in a pooled prevalence of frailty lower than that of general older population in Japan. Despite this possibility, the age-stratified meta-analyses suggest that prevalence of frailty is not necessarily lower in all age groups compared with other countries' populations: Japanese people aged 80 years or older are frailer than their international counterparts (Fig. 3 and a previous review^24^). Second, all studies included in the meta-analysis defined frailty using not the original version of the Fried criteria but versions with some modifications to some or all of the five components, apparently due to data availability. One example is that, while Fried et al. originally defined slow gait speed as being in the slowest 20% of usual walking speed stratified by gender and height, Shirooka et al. and Shimada et al. defined slow gait speed as a walking speed of less than 1.0 m/s. These modifications may have influenced the findings.^49^ Third, a high degree of heterogeneity was observed across the studies included in the meta-analysis (I^2^ = 79.2%, p < 0.001). This may be due to the effects of age distribution and/or gender proportion of the cohorts, since additional analyses stratifying the cohorts by age and gender alleviated the degree of heterogeneity in some groups. Fourth, a limited number of studies were included in this review, and the sample sizes of the studies were relatively small. Future research should include more studies with larger cohorts, especially for the oldest participants (85 years or older).

A major strength of this review is the robust methodology: the literature was comprehensively searched using 11 electronic databases; the identified studies were screened with standardized processes and assessed for heterogeneity, methodological quality, and publication bias; meta-analyses were repeated with stratification by age and gender. This review is the first to provide pooled evidence on the prevalence of frailty status among Japanese community-dwelling older people, and the findings are valuable and beneficial for clinicians, researchers, and policymakers. This information will help clinicians to treat older patients appropriately, stratifying the risks and life courses based on their frailty status. The overall prevalence of frailty among Japanese older people in the community is fundamental information for researchers and can be used to design population-based studies or randomized controlled trials, or to investigate pathophysiology or predictors of frailty. Policymakers may also use the information to effectively distribute limited health care resources and to translate the research findings into planning healthcare services.

In summary, this review has shown that the pooled prevalences of frailty, prefrailty, and robustness among Japanese community-dwelling older people were 7.5%, 48.1%, and 44.4%, respectively. These findings provide important basic information for all parties involved in Japanese frailty research. The age-stratified analysis showed the possibility that Japanese older people are less frail before their late 70's but frailer in later life than older people in other countries.

## Conflicts of interest

None declared.
